# More Efficient Complement Activation by Anti–Aquaporin-4 Compared With Anti–Myelin Oligodendrocyte Glycoprotein Antibodies

**DOI:** 10.1212/NXI.0000000000200059

**Published:** 2022-11-22

**Authors:** Magdalena Lerch, Kathrin Schanda, Eliott Lafon, Reinhard Würzner, Sara Mariotto, Alessandro Dinoto, Eva Maria Wendel, Christian Lechner, Harald Hegen, Kevin Rostásy, Thomas Berger, Doris Wilflingseder, Romana Höftberger, Markus Reindl

**Affiliations:** From the Clinical Department of Neurology (M.L., K.S., H.H., M.R.), Medical University of Innsbruck, Austria; Institute of Hygiene and Medical Microbiology (E.L., R.W., D.W.), Medical University of Innsbruck, Austria; Neurology Unit (S.M., A.D.), Department of Neuroscience, Biomedicine, and Movement Sciences, University of Verona, Italy; Department of Pediatric Neurology (E.M.W.), Olgahospital/Klinikum Stuttgart, Germany; Department of Pediatrics I (C.L.), Medical University of Innsbruck, Austria; Paediatric Neurology (K.R.), Witten/Herdecke University, Children's Hospital Datteln, Germany; Department of Neurology (T.B.), Medical University of Vienna, Austria; and Division of Neuropathology and Neurochemistry (C.L., R.H.), Department of Neurology, Medical University of Vienna, Austria.

## Abstract

**Background and Objectives:**

The objective was to study complement-mediated cytotoxicity induced by immunoglobulin G (IgG) anti–aquaporin-4 antibodies (AQP4-IgG) and anti–myelin oligodendrocyte glycoprotein antibodies (MOG-IgG) in human serum samples from patients suffering from the rare demyelinating diseases of the CNS neuromyelitis optica spectrum disorder (NMOSD) and MOG-IgG–associated disease (MOGAD).

**Methods:**

A cell-based assay with HEK293A cells expressing different MOG isoforms (MOGα_1-3_β_1-3_) or AQP4-M23 was used. Cells were incubated with human MOG-IgG or AQP4-IgG–positive serum samples together with active or heat-inactivated human complement, and complement-dependent cytotoxicity (CDC) was measured with a lactate dehydrogenase assay. To further quantify antibody-mediated cell damage, formation of the terminal complement complex (TCC) was analyzed by flow cytometry. In addition, immunocytochemistry of the TCC and complement component 3 (C3) was performed.

**Results:**

AQP4-IgG–positive serum samples induced higher CDC and TCC levels than MOG-IgG–positive sera. Notably, both showed a correlation between antibody titers and CDC and also between titers and TCC levels. In addition, all 6 MOG isoforms tested (MOGα_1-3_β_1-3_) could induce at least some CDC; however, the strongest MOG-IgG–induced CDC levels were found on MOGα_1_, MOGα_3_, and MOGβ_1_. Different MOG-IgG binding patterns regarding recognition of different MOG isoforms were investigated, and it was found that MOG-IgG recognizing all 6 isoforms again induced highest CDC levels on MOGα_1_ and MOGβ_1_. Furthermore, surface staining of TCC and C3 revealed positive staining on all 6 MOG isoforms tested, as well as on AQP4-M23.

**Discussion:**

Both MOG-IgG and AQP4-IgG are able to induce CDC in a titer-dependent manner. However, AQP4-IgG showed markedly higher levels of CDC compared with MOG in vitro on target cells. This further highlights the role of complement in AQP4-IgG–mediated disease and diminishes the importance of complement activation in MOG-IgG–mediated autoimmune disease.

Autoantibody-mediated autoimmune diseases comprise a broad spectrum that is rapidly evolving.^1^ Immunoglobulin G (IgG) antibodies against aquaporin-4 (AQP4-IgG) are found in most patients with neuromyelitis optica spectrum disorder (NMOSD), whereas antibodies against myelin oligodendrocyte glycoprotein (MOG-IgG) are associated with MOG-IgG–associated disease (MOGAD).^2-5^ Both conditions are rare demyelinating disorders of the CNS, which have distinct clinical, neuroradiologic, neuropathologic, and pathophysiologic features than multiple sclerosis (MS).

Autoantibodies can mediate their pathogenic potential through different immunologic pathways, some of which are activation of the complement system leading to complement-dependent cytotoxicity (CDC) through the formation of the terminal complement complex (TCC), internalization of antigen-antibody complexes, direct agonism/antagonism on a receptor, interruption of protein interactions, and antibody-dependent cellular cytotoxicity (ADCC).^1,6,7^ Most AQP4-IgG and MOG-IgG gain access to the CNS parenchyma through a disrupted blood-brain barrier.^4,8^

The pathogenic role of AQP4-IgG has been well studied and is characterized by activation of the complement pathway, astrocytopathy with loss of AQP4, and damage of oligodendrocytes and neurons.^3,9-17^ Activation of the classical complement pathway (CP) is a result from the interaction of C1q with orthogonal arrays of particles formed by the shorter AQP4-M23 isoform, which ultimately leads to TCC formation and consecutive cell damage.^18,19^ In 2019, eculizumab (anti-C5 antibody) was approved by the FDA and EMA as a treatment option for AQP4-IgG–positive NMOSD, highlighting the importance of CDC in the disease.^20^

By contrast, the role of complement activation in MOGAD is yet to be fully determined. Studies have shown the variable ability of MOG-IgG to activate complement in different experimental approaches.^21-23^ However, 2 neuropathologic studies observed complement deposition only in a subset of patients.^24,25^ Other mechanisms for the pathogenicity of MOG-IgG were also discussed.^26-29^ In addition, it has been shown that most patient-derived MOG-IgG require bivalent binding to their antigen, which disfavors C1q binding.^8,30^

In a recent study, we have shown that there are distinct binding patterns of human MOG-IgG to 6 major MOG isoforms (MOGα_1-3_β_1-3_), suggesting that the role of MOG-IgG in disease pathogenesis is even more complex.^31^ Here, we aimed to compare the ability of human serum samples with MOG-IgG or AQP4-IgG to induce complement-mediated cell damage in vitro.

## Methods

### Patient Serum Samples

In this cross-sectional study, we included 107 serum samples from patients with antibody-associated demyelinating diseases, of whom 68 were positive for MOG-IgG and 39 patients were positive for AQP4-IgG. Samples were obtained from 3 laboratories (Innsbruck and Vienna, Austria; Verona, Italy). All samples were analyzed for antibody positivity with either full-length MOGα_1_ or AQP4-M23 in 2 immunofluorescence live cell-based assays as described previously,^21,32,33^ where the cutoff titer value for MOG-IgG positivity was ≥1:160 and for AQP4-IgG positivity was ≥1:20. Further dilutions of sera were made to determine the antibody titer of each sample. Furthermore, all MOG-IgG–positive samples were tested for their binding to the 6 major MOG isoforms (MOGα_1-3_ and MOGβ_1-3_) to determine their respective binding pattern, as described recently.^31^ Demographic, serologic, and clinical data of patients are shown in Table and eTables 1, 2, and 3 (links.lww.com/NXI/A767).

**Table:**
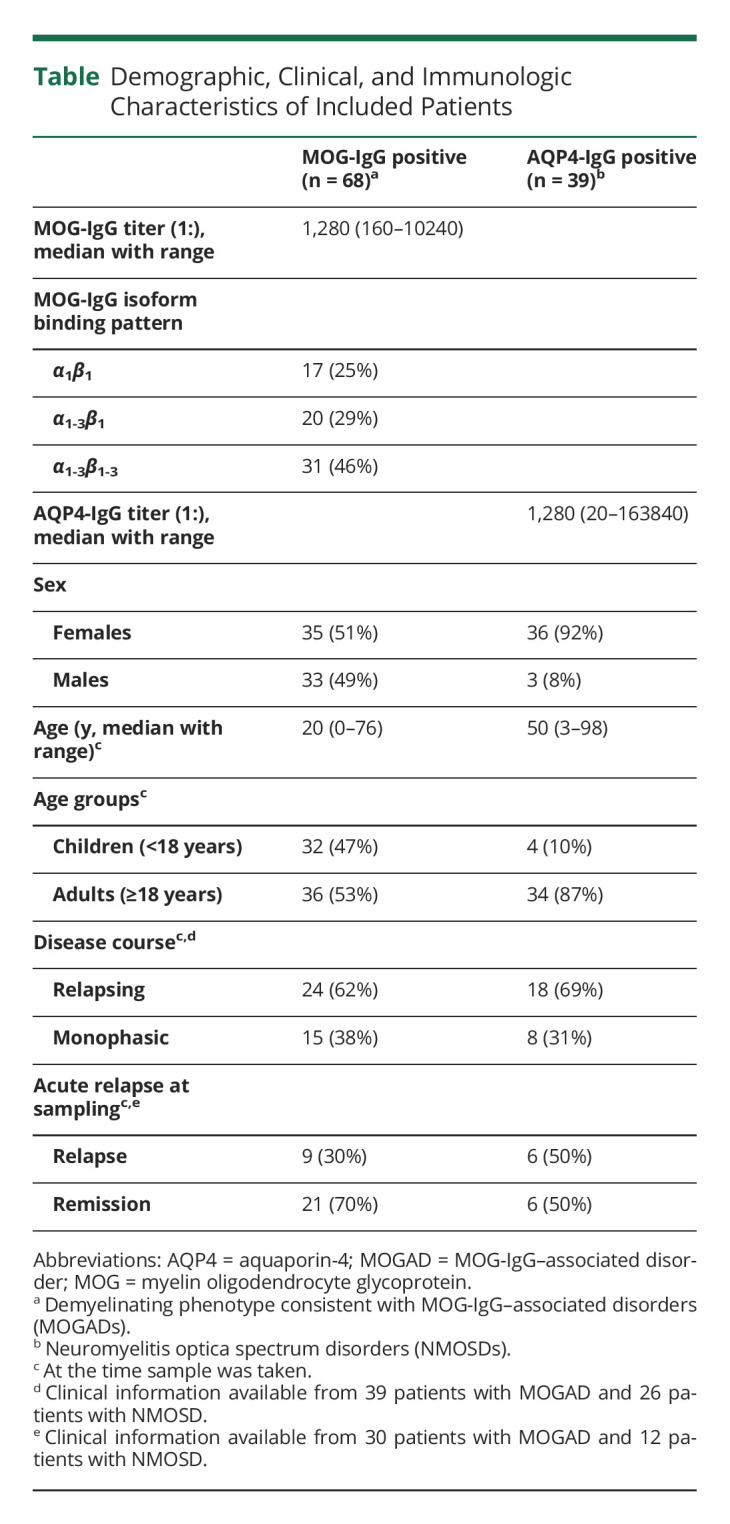
Demographic, Clinical, and Immunologic Characteristics of Included Patients

### Standard Protocol Approvals, Registrations, and Patient Consents

This study was approved by the ethical committees of the Medical University of Innsbruck, Austria (AM3041A and AM4059) and Medical University of Vienna (EK 1636/2019 and 1123/2015). Forty samples were obtained from the Neuropathology-Verona biobank. All patients or their caregivers gave written informed consent. All samples from participating centers were anonymized before sending them to Innsbruck, Austria.

### Lactate Dehydrogenase Cytotoxicity Assay

For the determination of CDC, a lactate dehydrogenase (LDH) assay was used (CytoTox 96 Nonradioactive Cytotoxicity Assay, Promega, Madison, WI) to measure the activity of LDH that was released into the cell culture medium after cell lysis,^18^ according to the manufacturer's instructions. Briefly, HEK293A (ATCC; LGC Standards GmbH, Wesel, Germany) cells were transfected with different MOG isoform constructs (MOGα_1-3_ and MOGβ_1-3_ either fused to enhanced green fluorescent protein (EGFP) or without tag, referred to as STOP; sequence variants of MOGα_1_: N30Q, P42S, E64K, A75S, R86Q, and H103A + S104E) in the pEGFP-N1 vector (Takara Bio USA, San Jose, CA) or with AQP4-M23 fused to emerald green fluorescent protein (EmGFP) in the Vivid Colours pcDNA 6.2-EmGFP vector (Thermo Fisher Scientific, Waltham, MA) with polyethylenimine (Sigma, St. Louis, Missouri). Detailed information on the MOG and AQP4 constructs was published elsewhere.^22,31,34^ After 24 hours or 48 hours, for MOG and AQP4, respectively, cells were washed 3 times with X-VIVO 15 media (Lonza, Basel, Switzerland) and incubated with 10% heat-inactivated serum samples (heat inactivation for 45 minutes at 56°C) together with 40% active or heat-inactivated (for 45 minutes at 56°C) human complement (Sigma) or factor B-depleted human serum (only used when stated; Quidel, San Diego, CA) in X-VIVO 15 media. Cells were incubated for 90 minutes at 37°C. To determine the maximal cell lysis on each plate, 10% lysis solution (part of the LDH assay kit, Promega) in X-VIVO 15 was added to control wells after 45 minutes. Next, cells were centrifuged for 5 minutes at 500*g*, and the supernatant was transferred to a 96-well plate (Thermo Fisher Scientific). Subsequently, reaction solution was added, and plates were incubated in the dark for 30 minutes. The reaction was stopped with stop solution, and absorption was measured at 492 nm (DTX880; Beckman Coulter, Brea, CA). Each serum was measured in duplicates for both active and inactivated complement, and all MOG isoforms were tested on the same day to avoid repeated thaw and freezing cycles. The amount of CDC was calculated as the difference of absorption between sera incubated with active complement and sera incubated with inactivated complement (to correct for unspecific differences in the endogenous LDH activity of the sera themselves) and is given in % to lysis buffer for each experiment. The cutoff values for MOG and AQP4 were calculated as the mean plus 2 times the SD of X-VIVO 15 together with complement and were run together with every independent transfection (MOG: n = 66, AQP4: n = 6).

We also used monoclonal humanized anti-MOG 8-18-C5^22^ (rh8-18-C5) and mouse anti-AQP4 E5415A^35^ (E5415A; produced in-house using E5415A-1H6-68 Hybridoma cells according to the manufacturer's instructions; #RCB4883, Riken, Tsukuba-shi, Ibaraki, Japan) as positive controls on MOG- and AQP4-transfected cells, respectively. Furthermore, Fab and F(ab')_2_ fragments of rh8-18-C5 were produced using the Pierce F(ab')_2_ and the Pierce Fab Micro Preparation Kits according to the manufacturer's instructions (Thermo Fisher).

### Analysis of Complement Products by Flow Cytometry and Immunocytochemistry

Complement activation product TCC was quantified by flow cytometry using an anti-C9neo antibody (clone WU13-15, Hycult Biotech, Uden, The Netherlands; 5 µg/mL) for both AQP4- and MOG-expressing cells. Furthermore, the cell surface deposition of TCC and C3 (clone 10C7, Cedarlane, Burlington, ON, Canada; 7.5 µg/mL) was assessed by immunocytochemistry. A detailed description of the methods is given in the Supplementary information (links.lww.com/NXI/A767).

### Statistics

Statistical analyses were performed using GraphPad Prism 9.1 (GraphPad Software Inc., La Jolla, CA) and SPSS 26 (IBM SPSS Statistics, Armonk, NY: IBM Corp). Comparison of complement activation between MOGα_1_ and AQP4 was examined with the Mann-Whitney test, and correlations were analyzed with the Spearman test. A Fisher exact test was used for calculating the OR for above cutoff positive sera. A Friedman test with post hoc Dunn was used for the comparison of CDC between MOG isoforms. For calculating the difference between expression vectors and MOG and AQP4 constructs, a Wilcoxon test was applied, and two-way ANOVA with the post hoc Šídák test was used for the analysis of CDC on MOG-EGFP vs MOG-STOP constructs. A four-parameter nonlinear least-squares fit regression was applied to calculate EC_50_ values of the concentration-dependent CDC response of rh8-18-C5 and E5415A. EC_50_ values were compared using Brown-Forsythe and Welch ANOVA with post hoc Dunnett. Two-way ANOVA with post hoc Tukey was used for the analysis of rh8-18-C5 Fab and F(ab')_2_ fragments and for MOGα_1_ sequence variant comparisons. To compare the CDC of the 3 MOG-IgG binding patterns on different MOG isoforms, we used the Kruskal-Wallis test with post hoc Dunn, and resulting *p* values were adjusted for multiple comparisons. Predictive factors for CDC were analyzed using multivariate regression analysis. Hodges-Lehmann median differences and Woolf's logit OR were used to assess the influence of disease course and acute relapses on CDC.

### Data Availability

The data used in this manuscript are available from the corresponding author after reasonable request or are included within this paper.

## Results

### Human AQP4-IgG Activates the Complement System Stronger Than MOG-IgG

**Figure 1:**
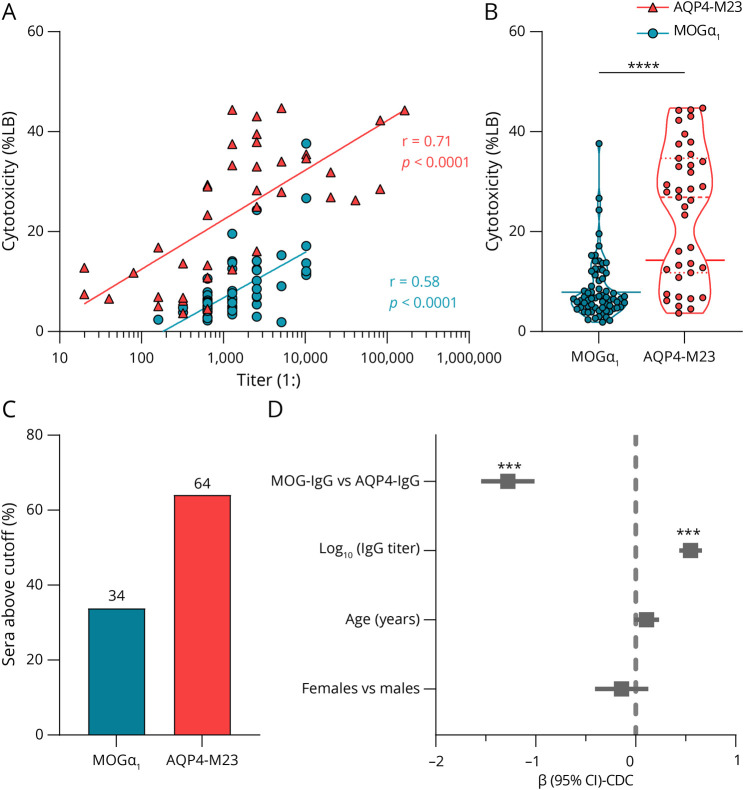
AQP4-IgG–Positive Serum Samples Show Stronger Complement Activation Than MOG-IgG–Positive Serum Samples (A) CDC (shown as percentage of lysis buffer determined by the LDH assay) of 68 MOG-IgG–positive human serum samples (blue circles; MOGα_1_-EGFP–transfected cells) and 39 AQP4-IgG–positive human serum samples (red triangles; AQP4-M23-EmGFP–transfected cells) is plotted against the antibody titers. Semi-log linear regression lines are shown. Spearman correlation: MOG: *r* = 0.58, *p* < 0.0001; AQP4: *r* = 0.71, *p* < 0.0001. (B) Violin plot (dashed line: median, dotted lines: quartiles) of all MOG-IgG (blue; n = 68) and AQP4-IgG (red; n = 39) positive serum samples tested is shown. Asterisks indicate the statistical difference of a Mann-Whitney test: ****p < 0.0001. The solid lines indicate the cutoff levels (mean + 2SD of cells treated with complement alone). (C) Percentage of MOG-IgG (blue) or AQP4-IgG (red) positive samples above the cutoff level (mean + 2SD of cells treated with complement alone) is demonstrated as bar graph. (D) The predictive role of MOG-IgG and AQP4-IgG antibody titers and status, age, and sex on complement-dependent cell lysis were analyzed by multivariate regression (R = 0.827, R^2^ = 0.684, F = 53.1, *p* < 0.001). Standardized regression coefficient beta is shown with 95% CI. ***p < 0.001. AQP4 = aquaporin-4; CDC = complement-dependent cytotoxicity; LB = lysis buffer; MOG = myelin oligodendrocyte glycoprotein.

We tested 39 serum samples positive for AQP4-IgG from patients with NMOSD and 68 serum samples positive for MOG-IgG from patients with MOGAD for their ability to activate the complement system. We used a cell-based LDH assay with HEK293A cells expressing different MOG isoforms fused with an EGFP tag or AQP4-M23 with an EmGFP tag and incubation of human sera together with active or inactivated human complement. Transfection efficiencies were slightly different between these expression vectors. However, they showed similarly specific surface binding of the monoclonal 8-18-C5 and E5415A antibodies for MOG and AQP4, respectively (eFigure 1, links.lww.com/NXI/A767; see also ref. 31). Antibody titers ranged from 1:160-10240 (median 1:1,280) in MOG-IgG–positive serum samples and from 1:20-163,840 (median 1:1,280) in AQP4-IgG–positive serum samples. Both showed a titer dependence of CDC (in % of lysis buffer; Spearman *r* for MOGα_1_: 0.58, *p* < 0.0001; Spearman *r* for AQP4: 0.71, *p* < 0.0001; Figure 1A). Importantly, the AQP4-IgG–positive serum samples showed an overall higher median cytotoxicity of 26.9% (to lysis buffer; 25–75th percentile 11.8–34.7) compared with MOG-IgG–positive serum samples with a median of 6.5% (25–75th percentile 4.8–11.1) (Mann-Whitney test: *p* < 0.0001, Figure 1B). To exclude the possibility that the EGFP tag could enhance the C1q-IgG interaction through clustering of MOG on the cell surface, we also tested MOG-IgG–positive serum samples on cells transfected with MOG-EGFP vs MOG without tag (referred to as MOG-STOP) and found lower CDC for MOGα1-EGFP and MOGβ1-EGFP compared with MOG variants without EGFP (eFigure 2C). Next, we evaluated the cutoff values for both proteins by calculating the mean plus 2 times the SD of transfected cells treated with complement alone (cutoff MOG: 7.9; cutoff AQP4-M23: 14.3) and found that for AQP4-M23 64% of sera tested were above the cutoff value compared with 34% for MOGα_1_ (Figure 1C) (Fisher exact test: *p* = 0.0044; OR = 3.5, 95% CI: 1.5–8.2). Using multivariate linear regression analysis, predictive values for CDC were significantly lower for MOG-IgG than for AQP4-IgG and were predicted by antibody titer (Figure 1D). In addition, CDC levels adjusted for antibody titers were significantly higher for AQP4-IgG (median 7.5, 25–75th percentile 4.7–9.8) compared with MOG-IgG (median 2.1, 25–75^th^ percentile 1.6–3.0, Mann-Whitney test: *p* < 0.0001).

To show that the complement activation observed was specific for a certain antigen and not directed against the cells themselves, we compared complement activation on HEK293A cells transfected with the expression vectors (pEGFP-N1 for MOG and pcDNA 6.2-EmGFP for AQP4) and could not find significant LDH activities in the media of the treated cells (eFigure 2, A and B, links.lww.com/NXI/A767). Although we used HEK293A cells for all our assays, we studied the expression of 2 complement inhibitors (CD46 and CD59) after transfection with MOGα_1_ or AQP4-M23.^36^ We observed increased CD59 expression of the transfected cells compared with nontransfected cells. However, expression levels were comparable between MOG- and AQP4-expressing cells (eFigure 2D). We also investigated the monoclonal anti-MOG antibody rh8-18-C5 and anti-AQP4 antibody E5415A for their ability to activate the complement pathway as a positive control (eFigure 3, A and B). These data showed that the rh8-18-C5 was able to induce higher maximal CDC values to MOG, but starting at higher concentrations, than the E5415A antibody to AQP4-M23 (Welch test comparison of EC_50_ values: ns, eFigure 3C), but this could also be due to the fact that the E5415A is completely of mouse origin. However, after incubation with rh8-18-C5 Fab and F(ab')_2_ fragments, there was no leakage of LDH into the media, showing the necessity of a Fc region for complement activation (eFigure 4A), and in addition, we studied sequence variants of the extracellular domain of MOGα_1_ to further underline the specificity of the assay. As expected, the H103A/S104E sequence variant (containing the rh8-18-C5 binding motif^31^) was not able to induce CDC, as shown in eFigure 4B. Finally, we found no clinical associations of CDC and antibody titers with acute relapses and disease course (eTable 4, links.lww.com/NXI/A767).

### Comparison of Complement Activation on Different MOG Isoforms

**Figure 2:**
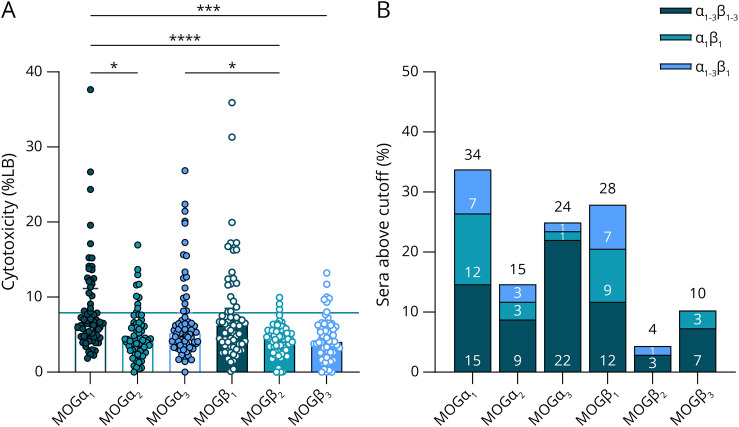
Complement Activation on Different MOG Isoforms (A) Comparison of CDC (as percentage of lysis buffer) of MOG-IgG–positive serum samples (n = 68) with 6 different MOG isoforms (MOGα_1-3_ and MOGβ_1-3_) is shown as a scatter dot plot. Bars indicate medians with interquartile range (Friedman test with post hoc Dunn: *p < 0.05, ***p < 0.001, and ****p < 0.0001). The solid line indicates the cutoff level (mean + 2SD of cells treated with complement alone). (B) Shows the percentage of MOG-IgG–positive serum samples above the cutoff value (mean + 2SD of cells treated with complement alone) for the 6 MOG isoforms. The percentages are plotted according to their respective binding patterns (α_1_β_1_, α_1-3_β_1_, and α_1-3_β_1-3_; values are given within the bars). AQP4 = aquaporin-4; LB = lysis buffer; MOG = myelin oligodendrocyte glycoprotein.

Like many other eukaryotic genes, the human MOG gene undergoes extensive splicing, leading to the formation of different isoforms.^37^ Recently, we have reported different binding patterns of human MOG-IgG to the major human MOG isoforms (MOGα_1-3_ and MOGβ_1-3_), namely the α_1_β_1_ (only recognizing the longest α_1_ and β_1_ isoforms), α_1-3_β_1_ (recognizing all 3 α and β_1_ isoforms), and α_1-3_β_1-3_ (binding to all 6 isoforms) pattern.^31^ Therefore, we wanted to evaluate whether the major 6 MOG isoforms (MOGα_1-3_ and MOGβ_1-3_) differ in their ability to activate the complement system after antibody binding. We found that MOGα_1_, MOGα_3_, and MOGβ_1_ could activate the complement system better compared with the other isoforms, which is shown in Figure 2A. As a next step, we again calculated which percentage of serum samples was above the cutoff value for each isoform and found 34% for MOGα_1_, 15% for MOGα_2_, 24%for MOGα_3_, 28% for MOGβ_1_, 4% for MOGβ_2_, and 10% for MOGβ_3_ (Figure 2B). These findings are as expected because MOGα_1_ and MOGβ_1_ isoforms were also those most recognized by human MOG-IgG as demonstrated in a previous study.^31^ eFigure 3A (links.lww.com/NXI/A767) shows the CDC rh8-18-C5 concentration curves to the 6 MOG isoforms tested, and a comparison of the EC_50_ values revealed a lower EC_50_ for MOGα_3_ (EC_50_: 0.9; 95% CI: 0.6–1.2) compared with MOGα_1_ (EC_50_: 3.2; 95% CI: 2.2–4.3), β_1_ (EC_50_: 2.3; 95% CI: 1.7–2.9), and β_2_ (EC_50_: 4.1; 95% CI: 2.0–6.1) (Brown-Forsythe and Welch ANOVA with post hoc Dunnett: *p* = 0.0017, 0.0017, and 0.47, respectively). To analyze differences in CDC induction of IgG belonging to the different binding patterns mentioned above, we sorted the sera based on their respective binding behavior. We found no difference in LDH activity on the MOGα_1_ and MOGβ_1_ isoforms between distinct binding patterns (eFigure 5A and 6A). However, for MOGα_3_, there was a difference between the α_1-3_β_1-3_ and α_1_β_1_ and the α_1-3_β_1-3_ and α_1-3_β_1_ patterns (eFigure 5C).

### Both AQP4 and MOG Antibodies Lead to Deposition of the TCC and C3 on the Cell Surface

To further establish cytotoxicity of human MOG-IgG and AQP4-IgG in our cell-based system, we urged to study the formation and deposition of complement products on the surface of MOG- or AQP4-expressing cells treated with MOG-IgG– or AQP4-IgG–positive serum samples.

**Figure 3:**
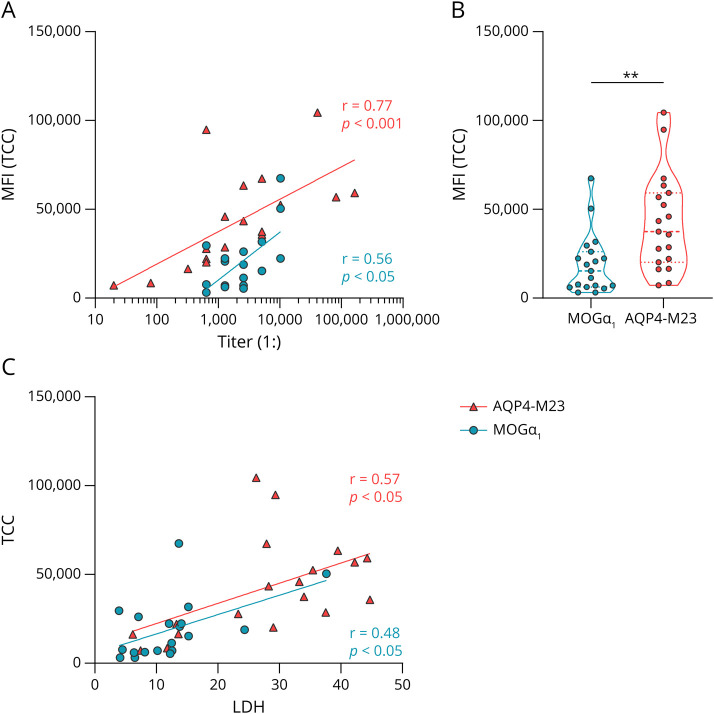
Quantification of TCC Deposition TCC deposition after complement activation of either MOGα_1_-EGFP (blue) or AQP4-EmGFP (red) transfected HEK293A cells by MOG-IgG or AQP4-IgG–positive serum samples together with human complement was quantified using an flow cytometry assay. (A) Mean fluorescence intensity (MFI) values are plotted against the antibody titers. The lines show semi-log linear regression. Spearman correlation: MOG: *r* = 0.56, *p* < 0.05, n = 19; AQP4: *r* = 0.77, *p* < 0.001, n = 19. (B) Comparison of MFI values between MOG (n = 19) and AQP4 (n = 19) is shown as violin plot (dashed line: median, dotted lines: quartiles). Mann-Whitney test: **p = 0.002. (C) The MFI values of TCC deposition of human sera are plotted against the respective values from the LDH assay (see Figure 1). Spearman correlation: MOG: *r* = 0.48, *p* < 0.05, n = 19; AQP4: *r* = 0.57, *p* < 0.05, n = 19. AQP4 = aquaporin-4; LDH = lactate dehydrogenase; MFI = mean fluorescence intensity; MOG = myelin oligodendrocyte glycoprotein; TCC = terminal complement complex.

For a confirmation of the higher values of AQP4-IgG–induced cell lysis compared with MOG-IgG, we quantified the formation of the TCC by flow cytometry. Therefore, HEK293A cells transfected with either MOGα_1_-EGFP or AQP4-M23-EmGFP were incubated with human heat-inactivated MOG-IgG (n = 19) or AQP4-IgG (n = 19) positive serum samples together with human complement. We found a correlation between antibody titers and TCC formation for both groups (Spearman *r* for MOGα_1_: 0.56, *p* = 0.012; Spearman *r* for AQP4-M23: 0.77, *p* < 0.001, Figure 3A). Importantly, incubation with AQP4-IgG again lead to a stronger activation of the complement cascade compared with MOG-IgG as shown in Figure 3B (TCC values of AQP4-M23: median 37,405, 25-75^th^ percentile 20,122–59,174; MOGα_1_: median 15,303, 25–75th percentile 6,207–26,145, *p* = 0.002). We also studied the correlation between TCC formation and cell lysis (LDH values) of the respective serum samples and found a correlation in the AQP4 group (Spearman *r*: 0.57, *p* = 0.011) and a trend toward correlation for MOG (Spearman *r*: 0.48, *p* = 0.04, Figure 3C).

In our immunocytochemistry experiments, human MOG-IgG and AQP4-IgG showed positive TCC staining together with active human complement but not after incubation with heat-inactivated complement on MOGα_1_- and AQP4-M23–expressing cells (eFigure 7, links.lww.com/NXI/A767). Cell morphology was clearly altered after complement activation, and many dead cells were present. Furthermore, TCC staining was present on cells expressing other MOG isoforms (α_2-3_, β_1-3_) after incubation with a human serum belonging to the α_1-3_β_1-3_ binding pattern (eFigure 9A) but was visible only on MOGα_1_ and MOGβ_1_ after incubation with a serum that shows an α_1_β_1_ binding pattern (eFigure 10A). However, treatment with a serum negative for MOG-IgG and AQP4-IgG did not result in TCC deposition (TCC; eFigure 13, A and C), and incubation of MOG-IgG or AQP4-IgG harboring sera on the transfection control cells showed no signs of complement activation (TCC; eFigure 13, B and D). As expected, the monoclonal antibodies rh8-18-C5 and E5415A were also able to activate the complement cascade, leading to TCC formation and deposition on cell surfaces (MOG isoforms, eFigure 11A; AQP4, eFigure 11B). As C3 is an important protein in the CP as well as in the alternative pathway (AP), we further wanted to investigate the extend of C3 cleavage after complement activation. Here, we found C3/C3b/iC3b staining for both MOG-IgG and AQP4-IgG after incubation with active complement but, interestingly, also with inactivated complement (eFigure 8). In addition, we observed positive staining after incubation with a representative α_1-3_β_1-3_ pattern MOG-IgG–positive serum on all MOG isoforms tested (eFigure 9B) and on MOGα_1_ and MOGβ_1_ after treatment with an α_1_β_1_ pattern serum but, to a much lesser extent, on the other isoforms (eFigure 10B). A small amount of C3b (and iC3) on cells treated with active complement was expected because the AP is always slightly active or because of failure of the CP to move forward to terminal activation.^38^ This was also observed after incubation with a representative double-negative serum (C3; eFigure 13, A and C) and after treatment with a MOG-IgG or AQP4-IgG–positive serum on the transfection controls (C3; eFigure 13, B and D) in combination with active complement. However, incubation with MOG-IgG or AQP4-IgG–positive serum samples without complement showed no positive staining (data not shown). Furthermore, as with TCC, the rh8-18-C5 and E5415A antibodies showed cleavage of C3 after incubation with active complement (MOG, eFigure 12A; AQP4, eFigure 12B). Finally, we used factor B-depleted serum to study the impact of the AP in our LDH assay and observed reduced cytotoxicity for MOGα_1_ and for AQP4-M23–transfected cells compared with normal human serum (eFigure 14).

## Discussion

We used a cell-based cytotoxicity assay to assess the ability of human serum samples positive for MOG-IgG or AQP4-IgG to activate the complement system on cells expressing different MOG isoforms or AQP4-M23, respectively. A cell-based assay was chosen because they are state of the art for the detection of MOG-IgG and AQP4-IgG in serum samples as it enables the binding to natively folded MOG or AQP4.^32,33^ Furthermore, cell-based assays were also used in former studies investigating CDC in AQP4-IgG– or MOG-IgG–mediated disease in vitro.^18,21,39-41^

We found increased LDH activity, released from damaged cells into the surrounding media, after incubation of serum samples together with active complement and could show that, in both cases, CDC was dependent on the MOG-IgG or AQP4-IgG titers but was absent on HEK293A cells lacking the respective antigen. A recently published article also found a correlation between AQP4-IgG titers and CDC; thus, we could confirm this association, but both studies showed no correlation with the clinical activity of patients.^41^ The role of complement activation in NMOSD is well established.^3,9-18^ Complement depositions have been observed in human AQP4-IgG–induced lesions in Lewis rats^14^ and in mice after injection of AQP4-IgG together with human complement into the brain.^15^ Furthermore, in studies investigating the neuropathology in patients with NMOSD, activated complement deposits were observed in human brain material.^11,12^ By contrast, in MOGAD, the role of complement is less clear, and studies made different observations.^21,22,24-26^ One study injected human MOG-IgG together with human complement into mouse brains and found only little complement activation,^26^ and furthermore, another investigation showed that only a portion of mouse monoclonal antibodies against MOG is able to induce demyelination in vivo.^42^ In an ex vivo mouse study, complement activation was only observed in 1 of 10 samples after treatment with purified human MOG-IgG, and this sample did not induce pathogenic lesions in vivo.^22^ Investigation of human MOG-IgG in vivo is hampered by the fact that a portion of human MOG-IgG does not cross-react with rodent MOG, or show reduced binding compared with human MOG.^22,23,43^ Of interest, one study showed that on transfer of cross-reactive human MOG-IgG into a rat model with MOG-specific T cells, T-cell infiltration was enhanced, but only traces of TCC were observed, whereas together with MBP-specific T cells MOG-IgG induced complement activation, leading to TCC deposition.^23^ Of note, recent neuropathologic studies investigating human brain material found either prominent^24^ or only sparse activated complement products.^25^ However, TCC deposition in MOGAD lesions does not necessarily mean that it is caused by MOG-IgG alone, and a synergistic action of humoral and cellular immune mechanisms may have an important impact on the formation of TCC deposits in a subset of patients.^23^

Here, we could confirm the importance of complement activation in human AQP4-IgG–provoked pathogenicity compared with human MOG-IgG because higher CDC levels were observed after incubation of sera harboring AQP4-IgG in the presence of active complement on AQP4-M23–expressing cells. Furthermore, after subtraction of serum samples that showed CDC levels lower than the control cutoff, we found that only 34% of MOG-IgG–positive serum samples were able to induce CDC compared with 64% of AQP4-IgG–positive serum samples. A recently published study found significantly higher levels of soluble activated complement products in patients with MOGAD compared with patients with NMOSD and MS.^44^ However, the study failed to detect significant levels of complement products in patients with NMOSD, where the role of complement is well established, and the high levels of complement products in MOGAD could also be due to a preceding systemic inflammation.^45^ In our study, we did not find a correlation between CDC and disease course or relapse. One study found such a correlation after investigating the CSF soluble C5a levels in patients with NMOSD,^46^ whereas another investigation showed that AQP4-IgG and CDC lack predictive and prognostic utility in patients with NMOSD.^47^

We also conducted additional experiments to further evaluate the ability of human MOG-IgG to activate the complement cascade on 6 different MOG isoforms (MOGα_1-3_ and MOGβ_1-3_). A recent study observed that the hydrophobic intracellular domain (which is only present in MOGα_1_ and MOGβ_1_) is necessary for antigen binding for most MOG-IgG and argued that this domain keeps MOG proteins separated at a certain distance to enable bivalent antibody binding,^30^ which is known to poorly interact with C1.^48^ Here, we also observed that the highest CDC responses were found against the 2 longest isoforms MOGα_1_ and MOGβ_1_. However, all 6 isoforms tested were able to trigger at least some complement activation after incubation with human MOG-IgG–positive serum samples and active complement. Moreover, MOG-IgG recognizing all 6 MOG isoforms^31^ showed the highest LDH activity on MOGα_1_ and MOGβ_1_. Of interest, we observed a difference between CDC of the 3 binding patterns (α_1_β_1_, α_1-3_β_1_, and α_1-3_β_1-3_) only on MOGα_3_, and furthermore, the EC_50_ value of the rh8-18-C5 monoclonal antibody on MOGα_3_ was lower when compared with MOGα_1_ and MOGβ_1_, which suggests an even more complex dynamic of complement activation on different MOG isoforms. To further investigate complement activation at a cellular level, we analyzed the presence of TCC and C3 on MOG- or AQP4-M23–expressing cells after initialization of the complement system through MOG-IgG or AQP4-IgG. IgG (more specifically IgG1 and 3 and relatively poorly IgG2) activate the complement system through the CP, which is conveyed by binding of C1q to a hexamer of surface bound IgG followed by formation of the CP C3 convertase that cleaves C3 into C3a and C3b. The latter binds to the surface of the target cell and mediates further activation, which ultimately leads to TCC formation. However, the AP serves as an amplification loop by generating the AP C3 convertase and is activated through a tick-over that constantly takes place and is tightly regulated.^7,38,49^ In our flow cytometry experiments, we found a correlation between TCC levels and antibody titers for both MOG and AQP4. The overall TCC level was higher for AQP4 compared with MOG, again demonstrating the more efficient complement activation through human AQP4-IgG.

We also observed positive C9neo staining on the surface of HEK293 cells expressing MOG isoforms or AQP4-M23 after treatment with human sera harboring the respective IgG together with active complement, but after incubation with heat-inactivated complement no staining could be observed visually. Again, we found visible TCC deposition on all 6 MOG isoforms tested. Of interest, C3 positive staining was visible after incubation with active and with heat-inactivated complement but only if human MOG-IgG or AQP4-IgG containing serum samples was present. A possible explanation would be the detection of iC3b, which is a degradation product of C3b, because the anti-C3 antibody used also binds to iC3b. Furthermore, other degradation products of C3b have been detected also in heat-inactivated sera.^50^ Moreover, C3b is also able to bind directly on IgG heavy chains, which could also lead to a positive staining in our setting.^7^ However, in all experiments conducted with active complement, we found a slight positive staining for C3, even without any IgG, which is explained by the constant activation of the AP. This is also supported by the reduced cytotoxicity observed after incubation with factor B-depleted serum in both AQP4- and MOG-expressing cells.

A recent study used a triple knockout cell line (CD46, CD55, and CD59 deficient) to study complement activation in patients with myasthenia gravis.^36^ We observed higher CD59 levels in the transfected cells compared with nontransfected cells; however, levels were comparable between MOG and AQP4 transfections. Despite the possible interference of CD59 with complement activation, we were able to measure CDC in our cell-based system and, importantly, used the same cell lines for AQP4 and MOG; we decided that this influence is negligible in our experimental setting.

Our study has several limitations. First, the sample size is small and limited by the amount of MOG-IgG– or AQP4-IgG–positive serum samples available, and the retrospective nature of the study is a restriction, too. Second, immunocytochemistry and flow cytometry experiments were only performed for representative serum probes. Third, whether human MOG-IgG induces CDC to MOG sequence variants would have also been interesting to study but was again limited by the available human material. In addition, a direct comparison of complement activation with both antigens in a system using primary oligodendrocytes and astrocytes or organoid-based experiments could lead to further insights into this important topic. Finally, purification of MOG- and AQP4-specific antibodies from patient sera would have been an interesting tool to normalize the antibody concentration for complement activation, but again this was limited by the amount of available patient serum samples.

To summarize, we demonstrated that human AQP4-IgG serum samples showed stronger activation of the complement system on AQP4-M23–expressing cells compared with MOG-IgG on MOG-expressing cells, but in both cases, this was correlated with the IgG titers. Furthermore, CDC was directed to all 6 MOG isoforms tested, but the strongest responses were detected to MOGα_1_, MOGα_3_ and MOGβ_1_. Together, these findings further underline the more pronounced role of complement activation in autoimmune diseases directed against AQP4 compared with MOG. Moreover, this study emphasizes the need for further studies to fully establish the role of complement activation in MOGAD including the potential use of complement inhibition as a therapeutic strategy.

## Glossary

ADCC: antibody-dependent cellular cytotoxicity
AP: alternative complement pathway
AQP4: aquaporin-4
C1, C3, C5: complement component 1, 3, 5
CDC: complement-dependent cytotoxicity
CP: classical complement pathway
IgG: Immunoglobulin G
LDH: lactate dehydrogenase
MOG: myelin oligodendrocyte glycoprotein
MOGAD: MOG-IgG–associated disease
MS: multiple sclerosis
NMOSD: neuromyelitis optica spectrum disorder
TCC: terminal complement complex
